# Pathways Between Parental Attitudes and Early Childhood Caries in Preschool Children

**DOI:** 10.3390/dj13050205

**Published:** 2025-05-02

**Authors:** Apolinaras Zaborskis, Aistė Kavaliauskienė, Jaunė Razmienė, Augustė Razmaitė, Vilija Andruškevičienė, Julija Narbutaitė, Eglė Aida Bendoraitienė

**Affiliations:** 1 Department of Preventive Medicine & Health Research Institute, Faculty of Public Health, Medical Academy, Lithuanian University of Health Sciences, 44307 Kaunas, Lithuania; 2 Department of Orthodontics, Faculty of Odontology, Medical Academy, Lithuanian University of Health Sciences, 44307 Kaunas, Lithuania; aiste.kavaliauskiene@lsmu.lt; 3 Department of Oral Health and Paediatric Dentistry, Faculty of Odontology, Medical Academy, Lithuanian University of Health Sciences, 44307 Kaunas, Lithuania; jaune.razmiene@lsmu.lt (J.R.); augurazm0210@lsmu.lt (A.R.); vilija.andruskeviciene@lsmu.lt (V.A.); julija.narbutaite@lsmu.lt (J.N.); egleaida.bendoraitiene@lsmu.lt (E.A.B.)

**Keywords:** children, parents, parental attitudes, childhood caries, toothbrushing, oral hygiene, pathway analysis

## Abstract

**Background/Objectives**: Parental attitudes play a crucial role in shaping children’s oral health habits and preventing dental diseases. This study aimed to explore the theoretical pathways through which parental behavior and attitudes toward child oral health can influence the dental caries experience as measured by the dmf-t index in preschool children in Lithuania. **Methods**: A cross-sectional study was conducted involving 302 children aged 4–7 years and their parents (262 mothers). Parental attitudes were assessed using the Parental Attitudes Towards Child Oral Health (PACOH) scale. For the children, the following variables were considered: sex, age, dental caries experience (dmf-t index in the primary dentition), oral hygiene index (Silness–Löe Plaque Index), toothbrushing frequency, and parental assistance with toothbrushing. Structural Equation Modeling (SEM) was applied for the data analysis. **Results**: The main path through which parental attitudes towards child oral health influenced the dmf-t index was via toothbrushing frequency (β = −0.17) or parental assistance with toothbrushing (β = 0.24). These factors were then linked to the oral hygiene index (β = 0.20 and β = −0.47, respectively), which ultimately influenced dmf-t (β = 0.52). The parents’ attitudes and toothbrushing frequency per se had no significant effect on children’s dmf-t (β = −0.06 and β = −0.04, respectively). The final model met all goodness-of-fit criteria: Chi-square test *p* = 0.211, Incremental Fit Index IFI = 0.994, Tucker–Lewis Index TLI = 0.982, Comparative Fit Index CFI = 0.994, and Root Mean Square Error of Approximation RMSEA = 0.038. **Conclusions**: Findings from this study demonstrate that parents play a significant role in determining children’s oral health. Regular toothbrushing, parental assistance with toothbrushing, and good oral hygiene are critical factors linking parents’ oral health-related attitudes to a child’s experience of early caries. Identifying the associations between dental caries risk factors helps plan interventions.

## 1. Introduction

The early childhood years (preschool age) are pivotal for children’s growth and development, yet children at this age cannot make their own decisions. Parents are decision makers in matters of children’s health and health care. Consequently, numerous life practices and studies have highlighted the crucial role of parents in promoting both children’s oral and overall health [1,2,3,4].

Most preschoolers struggle with proper toothbrushing and lack an understanding of the importance of this skill [5,6,7]. Parents, particularly mothers, play a crucial role in supporting their children’s oral health by providing guidance and assistance in toothbrushing and maintaining good oral hygiene [7,8,9]. Preschoolers’ diet depends heavily on their parents’ knowledge, attitudes, and eating habits within the family [10,11]. Parents are fully responsible for the timely first visit and subsequent regular visits to the dentist for dental check-ups, which are essential for the early detection and prevention of dental problems [12,13]. A study conducted across 17 countries, examining children aged three to four and their mothers, found notable differences in parental attitudes between children with dental caries and those without. [14]. Multivariate regression models confirmed that inadequate parental attitudes toward child diet and oral hygiene were risk indicators for dental caries in children [5,7,15,16]. Furthermore, as supported by the Social Learning Theories [17], when parents improve their own oral hygiene practice, their children accept these healthy behaviors more easily [10]. Therefore, parents serve as primary role models and educators for their children, demonstrating and promoting good oral hygiene habits and skills [18]. Poor oral hygiene in parents is considered a risk factor for both poor oral hygiene and dental caries in their children [19]. As such, parents are decision makers in matters of children’s oral health and health care; therefore, they should be regarded as the primary force in preventing oral diseases in children [5,20].

Based on the results of the studies considered above, it is clear that of all the parental factors, parents’ attitudes are particularly important. Literature reviews uniformly state that the children of parents with positive oral health attitudes had a lower caries prevalence compared to children of parents with poor oral health and attitudes [1,2,4]. It is also clear that parental factors can influence children’s oral health status through various oral health practices of children. Researchers identify regular toothbrushing, parental assistance with toothbrushing to maintain good oral hygiene, a diet that limits sugar consumption, and regular dental visits as the most important practices for the link between parental factors and children’s oral health status [1,2,4]. However, some studies suggest that parental factors can directly impact children’s oral health status [21,22,23]. Thus, although parental attitudes have been identified as an important factor and its association with various aspects of young children’s oral health has been shown, it is not sufficiently clear whether these parental attitudes lead to children’s oral health status directly or indirectly and what the paths, sequences, and weights of the associations between factors in various links of the complex pathway are.

An essential aspect of the methodology in studies of this nature is the selection of an appropriate instrument to assess parents’ knowledge, beliefs, and attitudes regarding preschool children’s oral health and hygiene practices. Various instruments have been developed and employed in previous cross-sectional studies for this purpose [24]. Among these, the instrument developed by Pine and Adair (2004) has received particular attention [14,25]. The theoretical framework of this instrument integrates three well-established models: the Theory of Planned Behavior [26], the Health Belief Model [27], and the Health Locus of Control Model [28]. It is a comprehensive questionnaire, with items specifically targeting parental beliefs and attitudes toward three core dimensions of children’s oral health: toothbrushing, sugar snacking, and dental caries [14]. This scale has been previously utilized in several studies [10,15,16,29,30]. Given its broad scope, we refer to it as the Parental Attitudes towards Child Oral Health (PACOH) scale. The instrument was translated into Lithuanian, validated, and applied to explore associations between parental attitudes and their ability to promote positive oral health behaviors in children [10,31]. In our recent study, we conducted a structural analysis of the PACOH scale and evaluated several abbreviated versions [32].

A complex pathway that simultaneously evaluates the association of multiple behavioral factors can be analyzed using Structural Equation Modeling (SEM) [33,34]. Several studies have already been conducted using this approach to examine preschoolers’ oral health. One such study, conducted by Qiu et al., explored factors related to dental caries in Chinese children aged 5 years [35]. The results showed that there is a consistent chain of relationships between oral health-related factors: socioeconomic factors had an impact on parents’ oral health knowledge, which influenced children’s oral health behavior through the impact of parents’ oral health attitudes, and, finally, children’s oral health behavior was directly linked to the presence of dental caries. The next complex model that simultaneously evaluated the multidimensional behavioral pathways was tested on Hong Kong preschool children aged 5–7 years [36]. The SEM analysis that was conducted in this study showed that a better socioeconomic status of the parents led to better oral health knowledge and attitudes and also to their children’s better oral health knowledge and behavior. The positive oral health behavior of children, in turn, led to better oral hygiene and health.

Despite the modern approach to analyzing behavioral data using SEM, we believe that both of the abovementioned models are limited in their consideration of parental behavioral factors. These include parental support in fostering children’s healthy lifestyle-related behaviors and parental responsibility for ensuring early and regular dental visits to monitor their children’s oral health, among others. Therefore, we decided to consider more parental factors related to children’s dental health.

**Figure 1:**
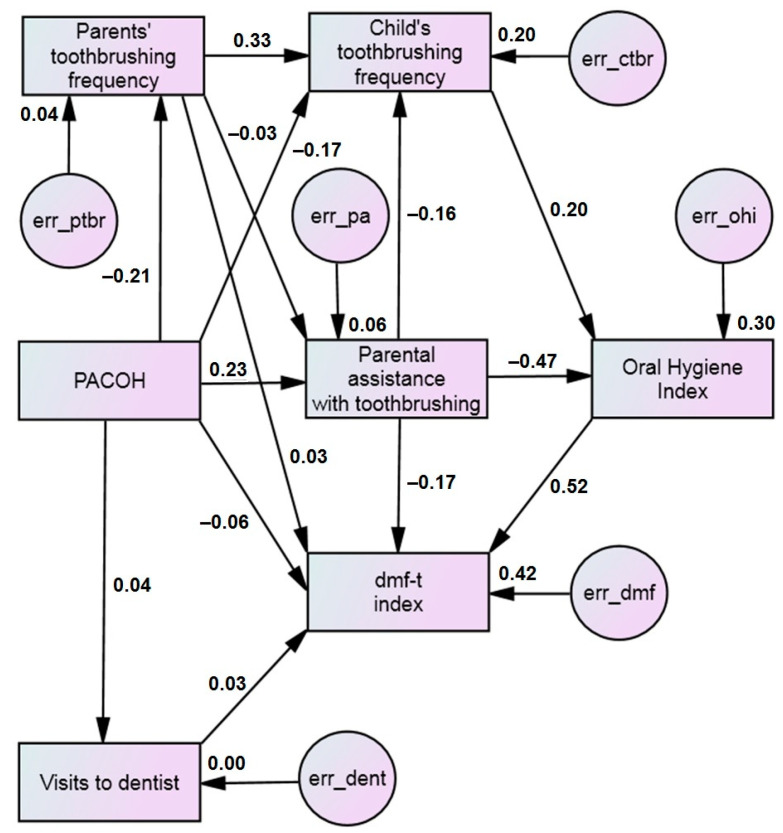
Path diagram of hypothesized relationships between parental attitudes toward child oral health (PACOH) and early childhood caries (dmf-t index)—full model. Notes: numbers on pathways are standardized regression coefficients; numbers next to boxes are squared multiple correlations; and err_xxx are errors of corresponding variables which represent unmeasured variables. For other comments see Results.

This study aimed to explore the theoretical pathways through which parental behaviors and attitudes toward child oral health can influence the dental caries experience as measured by the dmf-t index in children aged 4–7 years. It was planned to test the model among preschool children attending kindergartens in Lithuania (Kaunas region). Based on previous SEM models [35,36], as well as based on correlations between the predictors of child dental health found in other studies [5,7,13,15,16,22], we hypothesized a conceptual model presented in Figure 1. The specific objectives of our study were as follows: (1) to test whether parental behaviors and attitudes have a significant direct effect on child dental health; (2) to test which of the parental factors—parental oral health-related behavior or parental attitudes toward child oral health—has a greater impact on a child’s toothbrushing skills. In order to simplify the analysis, we did not include socio-demographic factors in the SEM model, as we analyzed their effect in another way.

## 2. Materials and Methods

### 2.1. Study Design, Participants, and Ethical Consideration

This study utilized an observational cross-sectional design. The target population consisted of preschool-aged children (4–7 years old) and their parents residing in the Kaunas region, Lithuania.

The sample size was estimated to ensure the reliability and validity of the results concerning children’s oral health, which was the focus of other parallel studies [28,29]. The calculation was based on a z-test to detect a 10% difference in caries prevalence between any two groups of children (e.g., between boys and girls), with a significance level of α = 0.05 and a power of 0.8. Assuming a 50% caries prevalence (the point of highest uncertainty), a minimum sample size of 305 participants was determined using G*Power 3.1 software (University of Düsseldorf, Düsseldorf, Germany) [37]. Considering that approximately 25% of parents might decline participation [31], it was decided to invite the parents of 425 children to take part in this study.

The required number of subjects was selected from six randomly chosen kindergartens in the Kaunas region (three from Kaunas city and three from a rural area). The subject’s inclusion criteria for participation in the study were parental consent for the child’s examination and an agreement to participate in the questionnaire survey, as well as the child’s willingness to undergo an oral health examination. No specific exclusion criteria related to children’s health were applied, since the selected kindergartens were attended by healthy children. Data collection took place from April to June 2023. Of the 425 parents invited, 307 participated, resulting in a 71% response rate. After data cleaning, 302 parent–child pairs were included in the final analysis.

This study was approved by the Kaunas (Lithuania) Regional Committee for Biomedical Research Ethics (No. 2023-BE-10-0003, issued on 7 March 2023). Written informed consent was obtained from all parents who agreed to participate in this study, as well as from the administrators of all kindergartens visited.

### 2.2. Questionnaire

The self-administered anonymous questionnaire was completed by either the father or the mother.

The questionnaire gathered socio-demographic information, including the child’s sex and age, the participating parent (1 = father, 2 = mother) and his/her education level (1 = less than college, 2 = college or university), household location (1 = urban area, 2 = rural area), and average total family income per month (in analyses it was dichotomized to 1 = ≤2700, 2 = > 2700 EUR/month). Respondents were then asked about their child’s toothbrushing habits. The frequency of toothbrushing was recorded as follows: 1 = at least twice a day, 2 = once a day, 3 = less often or irregularly, and 4 = never. Parents were also asked whether the child brushes their teeth independently (coded as 1) or if they assist with toothbrushing (coded as 2). Additionally, parents reported how often they themselves brush their teeth (using the same scale as for children) and how often the child visits the dentist for check-ups (1 = at least once a year (regularly), 2 = less often (irregularly)).

The questionnaire also included the 38-item PACOH scale [14,25]. Responses to the scale items were rated on a 4-point Likert scale, with the following scores: 1 = ‘strongly agree’, 2 = ‘agree’, 3 = ‘disagree’, and 4 = ‘strongly disagree’. If none of these response categories were selected, the item was assigned a value of 2.5. For statements where agreement indicated a positive attitude towards the child’s dental health, the response scores were inverted. The PACOH scale scores were then summed, with higher total scores representing a more positive respondent attitude. We used the Lithuanian version of the PACOH scale, which had been translated from English, validated, and applied in previous studies [10,31,32]. In this study, the internal reliability (Cronbach’s alpha) for the scale was 0.790.

### 2.3. Clinical Dental Examination

The clinical dental examination of the children was conducted in the kindergarten under standardized conditions, in accordance with WHO recommendations [38]. Parents received written information regarding their child’s oral health status.

The examinations were carried out using a standard explorer and mirror by trained and calibrated dentists. They evaluated caries experience and oral hygiene. Inter-examiner agreement (Kappa statistic) for the assessment of seven indicators of caries intensity ranged from 0.81 to 0.93, and for the assessment of oral hygiene it was 0.93.

The dmf-t score was used to evaluate dental caries experience, representing the total count of decayed (d), missing (m), and filled (f) primary teeth [38].

The oral hygiene (Silness–Löe Plaque) index scored plaque accumulation on a tooth surface: 0 = no plaque; 1 = a film of plaque adhering to the free gingival margin and adjacent areas of the tooth, but not visible to the naked eye (plaque can only be detected using a probe); 2 = moderate accumulation of plaque, visible to the naked eye along the gingival margin and adjacent areas; and 3 = heavy accumulation of plaque along the gingival margin and on the tooth surface. The plaque index is averaged by summing the scores and dividing by the number of teeth, and then is categorized as follows: 0 = excellent (no plaque), 0.1–0.9 = good (coded as 1), 1.0–1.9 = satisfactory (2), and 2.0–3.0 = poor (3) [39].

### 2.4. Data Analysis

Descriptive statistics were calculated to estimate the frequency (*n*), percentage (%), mean, standard deviation (SD), and range of the variables. Spearman’s correlation was calculated to evaluate the bivariate association between observed variables. The Chi-square test and z-tests were applied to compare percentages of categorical variables (e.g., oral hygiene status, toothbrushing frequency), while the *t*-test was used to assess differences in the means of continuous variables (e.g., sum score of the PACOH scale, dmf-t index). A *p*-value < 0.05 was considered statistically significant, and the confidence interval (CI) was set at 95%. These analyses were conducted using SPSS statistical software (version 21; IBM SPSS Inc., Chicago, IL, USA).

Using the path analysis methodology of Structural Equation Modeling (SEM) [33,34,40], we developed a model to assess the pathways through which parental attitudes toward child oral health influence the dental caries experience, as measured by the dmf-t index. The model examined the hypothesized causal relationships between variables identified a priori through correlation analysis. These relationships were considered to be unidirectional. In the model, the sum score of the PACOH scale was considered an exogenous variable, while children’s and parents’ toothbrushing frequencies, parental assistance with toothbrushing, OHI, and the dmf-t index were treated as endogenous variables, each containing error components. Structural Equation Modeling was performed to assess the final model using an unweighted least squares estimation method, appropriate for the categorical nature of the data [34,41]. The model provided standardized regression coefficients (β) to indicate the strength of associations between connected variables. Squared multiple correlations (R^2^) were reported for each endogenous variable, representing the proportion of variance explained by its predictors. An example of the path diagram is presented in the Results section.

The impact of socio-demographic factors on the model was examined using AMOS multi-group analysis. This approach tested the invariance of regression weights and other model estimates across groups of respondents. Differences in estimates were tested using the pairwise parameter comparisons feature [34].

Finally, model goodness-of-fit statistics were estimated. The χ^2^/degree of freedom (df) statistic was used to assess the discrepancy between the sample and fitted covariance matrix, where *p* > 0.05 indicated consistency between the model and the data. Model fit was further evaluated using the Incremental Fit Index (IFI), Tucker–Lewis Index (TLI), the Comparative Fit Index (CFI), and the Root Mean Square Error of Approximation (RMSEA). An IFI, TLI, and CFI > 0.9, along with an RMSEA < 0.09, indicated a good fit to the data [40,41]. Path analysis was conducted using AMOS 21 (SPSS Inc., Chicago, IL, USA, 2012) [34].

## 3. Results

### 3.1. Sample Characteristic

Table 1 presents the descriptive statistics for the socio-demographic and clinical variables used in this study. The sample included approximately equal proportions of boys and girls, as well as balanced proportions of preschoolers in the two age groups (<5 years and ≥5 years). The children’s ages ranged from 3.9 to 6.9 years, with a mean age of 5.18 (SD = 0.84) years. There were more mothers than fathers in the study, and respondents were more likely to have a college or university education.

Across the sample, on average, half of the respondents reported that they assist their children with brushing their teeth. Obviously, a higher percentage of parents in the younger children’s group provided this help compared to those in the older group (61.6% vs. 44.4%, *p* = 0.002). Overall, 55.3% of children and 75.5% of parents brushed their teeth regularly, defined as at least twice a day.

The clinical examination found that the majority of children (63.8%) had excellent or good dental hygiene, while a significant proportion (36.2%) had a thick layer of plaque on their teeth. On average, children had 3.64 caries-affected teeth. Table 2 illustrates how children’s dental caries experience, assessed by the mean of the dmf-t index, varies across the socio-demographic groups of the respondents. The data indicate that children in the older age group, as well as those whose parents have lower levels of education and income or who reside in rural areas, have significantly more teeth affected by caries.

### 3.2. Parental Attitudes

Parental attitudes toward child oral health were assessed using the PACOH scale, with the sum score representing the overall attitude. The one-sample Kolmogorov–Smirnov test retained the null hypothesis (*p* = 0.627), indicating that the distribution of the PACOH sum scores was normal, with a mean of 122.69 and a standard deviation of 10.00. The scores across the sample ranged from 95 (indicating the most negative attitude) to 147 (indicating the most positive attitude). The distribution of scores was consistent across groups based on children’s gender and age, as well as between fathers and mothers (Table 3). Respondents with higher levels of education, those living in the city, and those with higher family incomes tended to express more positive attitudes compared to their corresponding counterparts.

### 3.3. Correlation Between Study Variables

Table 4 shows the bivariate Spearman’s correlations between the study variables. The strongest correlation (ρ = 0.69) was observed between the oral hygiene index and the dmf-t index, indicating that poorer oral hygiene in children is associated with a higher number of teeth affected by caries. A significant negative correlation was also found between the sum scores of the PACOH scale and the dmf-t index (ρ = −0.21). The negative sign of this estimate shows that a more positive parental attitude toward the child’s oral health is linked to fewer early caries lesions in children, while the high significance of this relationship (*p* < 0.01) suggests a possible direct relationship between the variables. The calculated correlations also indicate that parental assistance with child’s toothbrushing is significantly associated with better child oral hygiene (lower OHI) (ρ = −0.52), a lower dmf-t index (ρ = −0.47), and regular dental visits (ρ = −0.17). It is also seen that children’s and parents’ toothbrushing frequencies are significantly and positively correlated (ρ = 0.37). The correlation between mothers’ and children’s toothbrushing frequency (ρ = 0.40, *p* < 0.01) was stronger than that between fathers and children (ρ = 0.26, *p* = 0.110).

### 3.4. The Path Model

**Figure 2:**
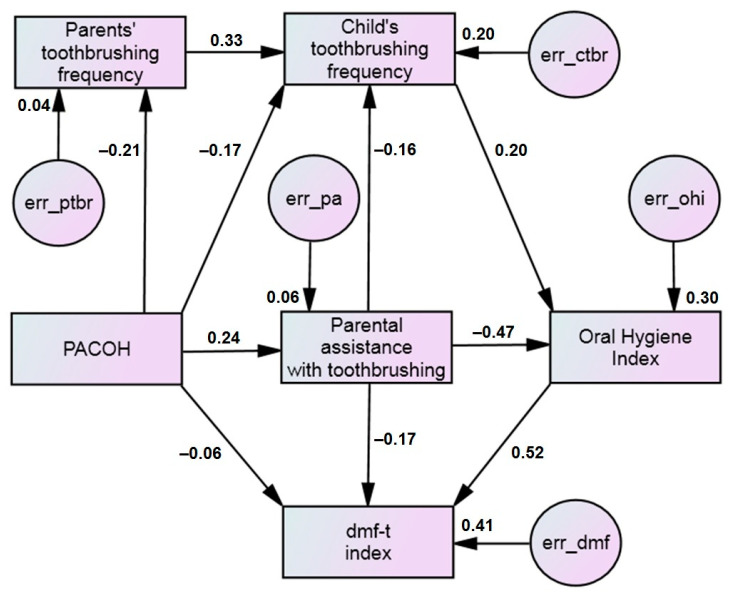
Path diagram of relationships between parental attitudes toward child oral health (PACOH) and early childhood caries (dmf-t index)—complete structural model. See notes for Figure 1.

To clarify the mechanisms underlying the relationships between the selected predictors of early dental caries, we used a path analysis. A hypothetical model illustrating the relationships between the study variables was already presented in Figure 1. This model demonstrated satisfactory goodness-of-fit statistics: Chi-square test *p* = 0.014, IFI = 0.972, TLI = 0.913, CFI = 0.971, and RMSEA = 0.071 (90% CI: 0.030–0.113). It highlights the following key findings. First, parental attitudes, as measured by the sum score of the PACOH scale, have an indirect relationship with the child’s dental caries lesions (dmf-t index) through several significant pathways. Second, some factors in the model exhibit only weak direct relationships with dental caries. To simplify the model and improve its overall quality, we removed these weak relationships, except the path from PACOH to dmf-t. Figure 2 presents the path diagram of the complete structural model. The goodness-of-fit statistics of this model perfectly met all the criteria: Chi-square test *p* = 0.211, IFI = 0.994, TLI = 0.932, CFI = 0.994, and RMSEA = 0.038 (90% CI: 0.000–0.095).

The primary pathway through which parental attitudes influenced the dmf-t index was via the child’s toothbrushing frequency (β = −0.17) and parental assistance with toothbrushing (β = 0.24). These factors were then linked to the oral hygiene index (OHI) (β = 0.20 and β = −0.47, respectively), which ultimately impacted the dmf-t index (β = 0.52). The direct effect of PACOH on dmf-t was minimal (β = −0.06). Overall, oral hygiene, parental assistance with toothbrushing, and PACOH—though the latter had a minimal effect—explained 41% of the variance in dmf-t values (the squared multiple correlation for this variable was R^2^ = 0.41). The significance of these estimates is presented in Table 4 (column ‘Total sample’).

The presented chain of relationships also includes parents’ toothbrushing frequency. This factor is also related to parental attitudes (β = −0.21). However, it appeared that the effect of this pathway on a child’s toothbrushing frequency is greater than the direct effect of the parental attitudes (β = 0.33 vs. β = −0.17).

### 3.5. Comparison of Path Model Between Socio-Demographic Groups

Because socio-demographic variables were not included in the path model, their effects were analyzed using the SEM multi-group analysis feature. The pairwise comparisons of the model parameters between socio-demographic groups revealed some differences in the model structure (Table 5).

When comparing respondent groups by the child’s gender, it was observed that in the girls’ group, the effect of the OHI on dmf-t was significantly stronger than in the boys’ group (β = 0.63 vs. β = 0.45, *p* = 0.051). On the other hand, in the boys’ group, positive parental attitudes were more strongly—and even statistically significantly—associated with lower levels of caries in children compared to the girls’ group (β = −0.28 vs. β = −0.02, *p* = 0.010). A comparison of the preschoolers’ age groups showed that among older children, the relationship between the toothbrushing frequency and oral hygiene status was stronger than among younger children (β = 0.30 vs. β = 0.10, *p* = 0.050).

Differences in the relationships between variables were also found when comparing fathers’ and mothers’ reports: the relationship between the toothbrushing frequency and oral hygiene status was stronger in mothers’ reports (β = 0.24 vs. β = −0.04, *p* = 0.029), while parental assistance with toothbrushing had a stronger direct effect on the dental health status in fathers’ reports (β = −0.46 vs. β = −0.13, *p* = 0.013). When comparing respondents by household location, it was found that parents’ attitudes had a stronger effect on their own toothbrushing frequency in rural areas than in urban areas (β = −0.33 vs. β = −0.08, *p* = 0.020), while parents’ attitudes on children’s toothbrushing frequency in rural areas was weaker than in urban areas (β = 0.09 vs. β = −0.21, *p* = 0.312).

The structural weights of the model were invariant when comparing the groups of respondents by education level and by the family’s income. However, in the group of respondents with a higher education, the selected predictors better explained the variance of the dmf-t index (R^2^ = 0.44 vs. R^2^ = 0.32). Additionally, squared multiple correlations were higher in the group of fathers’ reports compared to the group of mothers’ reports (R^2^ = 0.57 vs. R^2^ = 0.41).

## 4. Discussion

This study confirmed that parental attitudes toward children’s oral health are a significant factor associated with early dental caries in preschoolers aged 4–7 years. However, the direct relationship between parental attitudes and dental decay outcomes appeared to be weak. Instead, the findings suggest that this relationship is indirect, mediated by key factors such as regular toothbrushing, parental assistance with toothbrushing, and good oral hygiene. These factors play a crucial role in linking parental attitudes to a child’s experience of early caries. Additionally, this study highlighted the importance of a parental example—specifically, parents’ own regular toothbrushing—as a key influence on their children’s toothbrushing frequency. This model adequately fitted the data.

In the present study, the sample size of the parents and preschool children was sufficient to test the proposed hypotheses. The sample was representative of children attending kindergartens in the Kaunas region, with respect to both age and gender. Their mean dmft score was 3.64, which is significantly lower than the score of 5.56 reported in a comparable study conducted 12 years ago (2010/2011) [31]. Oral hygiene index scores were also found to have improved. These positive changes were associated with improved parental attitudes toward their children’s oral health care, as reflected by an increase in the total PACOH sum score from 112 to 122 [42]. However, despite these improvements, the dental health status of Lithuanian preschool children remains poor compared to their peers in other European countries [43], even though dental treatment is free for children up to 18 years of age and annual dental visits are mandated.

In this study, we applied the an SEM pathway analysis method to examine the direct and indirect effects of parental factors on children’s caries. This approach offers an advantage over multiple regression analysis, which can only identify direct relationships between variables and the outcome. Therefore, our study contributes to the growing body of research utilizing the SEM approach [35,36,44,45].

In line with the findings from the previous SEM analyses [35,36], as well as from other studies [5,20], our study refuted the possibility of a direct link between parental attitudes and childhood dental decay. Instead, a strong association was found between positive parental attitudes and the establishment of regular toothbrushing habits in children, which in turn is associated with better oral hygiene. And finally, and there is no debate about this, better oral hygiene in children ensures healthier teeth. Strong bivariate correlations between these factors were also shown in our correlation analysis. Such correlations have been described in many other studies [2,46,47,48,49].

The results of this study also revealed an alternative link between parental attitudes and children’s oral hygiene through parental assistance with toothbrushing. According to the American Academy of Pediatric Dentistry [50], as well as the Lithuanian Association of Dentists [51], it is recommended that parents should assist their young children with brushing their teeth, as younger children often lack the motivation and dexterity required to do so effectively [43]. In the present study, 53% of parents reported assisting their children with toothbrushing. This factor may have directly contributed to, or indirectly through the oral hygiene influence, the better dental health of the children in this study. This finding aligns with the results of previous studies [49,52,53].

One of the objectives of the present study was to examine which parental factors—either parental oral health-related behavior or parental attitudes toward child oral health—have a greater impact on a child’s toothbrushing skills. Based on the estimates from the complete structural model (Figure 2), it appeared that the effect of parents’ toothbrushing frequency on their child’s toothbrushing frequency was stronger than the direct effect of parental attitudes (β = 0.33 vs. β = −0.17). As the multi-group analysis (see below) showed, this difference was even more pronounced among rural residents. However, this comparison is not entirely accurate because the factors being compared were measured using different units. Additionally, parental attitudes influence a child’s behavior through the parents’ behavior, and this indirect effect is notable (−0.21 × 0.33 = −0.07). Even with these adjustments, it remains clear that parents’ oral health practices have a stronger influence than their attitudes on children’s oral health practices [36]. This finding suggests that preschool-aged children are more likely to imitate their parents’ oral health-related behaviors. Indeed, such behavior is consistent with the Social Learning Theories [17]. Research indicates that methods like observation, role modelling, and imitation—core elements of Social Learning Theory—can be effective in promoting positive behavior in preschool children [54].

In contrast to the studies conducted by Qiu et al. [35] and Zhang et al. [36], we chose not to include socio-demographic factors in the SEM model. This decision was not made to simplify the model, but rather to extract more detailed information from it. Instead, we applied an SEM multi-group analysis [34] to examine how the model parameters varied across different socio-demographic groups. Below are some examples.

A bivariate correlation analysis revealed that the correlation between the mothers’ and children’s toothbrushing frequency was stronger than the correlation between fathers and children. This observation aligns with findings from previous studies [36,45]. One possible explanation is that mothers are typically the primary caregivers during the early stages of a child’s life and therefore may play a more significant role in shaping their children’s oral health. In the multi-group analysis, the path from the mothers’ toothbrushing frequency to the children’s toothbrushing frequency was more pronounced than that from fathers to children, though the difference was not statistically significant (β = 0.35 vs. β = 0.21, *p* = 0.190). However, when comparing the relationship between children’s toothbrushing and their OHI across parents, we found that this relationship was significantly stronger in the mothers’ group (β = 0.24 vs. β = −0.04, *p* = 0.029). This may be explained by the fact that the experience of toothbrushing copied from mothers ensures better plaque removal. On the other hand, fathers’ assistance with children’s toothbrushing appears to have a stronger impact on oral hygiene and was significantly more strongly associated with better child dental health than the mothers’ assistance (β = −0.46 vs. β = −0.13, *p* = 0.018). This may be due to the novelty of the father’s involvement, which may enhance the child’s engagement, or to the stronger influence of the father as a role model, particularly for boys; children may also respond more easily to a father’s authority and therefore be more submissive [55].

Similarly, it can be observed that among girls, the impact of oral hygiene on dental health is stronger than among boys. Additionally, among children older than 5 years, regular toothbrushing has a greater effect on oral hygiene than among younger preschoolers. When comparing respondents by household location, it was found that in rural areas parents’ attitudes had a stronger effect on their own toothbrushing frequency, whereas their attitudes had a much weaker effect on children’s toothbrushing habits. This finding may help explain why children from rural areas had a higher number of caries-affected teeth. The poorer dental health of rural children, as reported in other studies [56,57], could also be attributed to limited access to dental care and preventive programs.

When comparing respondents by the education level and family income, we did not find significant differences in the relationships between variables, which contrasts with findings from similar studies [35,36,44,45]. However, in the group of respondents with a higher education, the model showed that the analyzed predictors had a greater overall effect on the variance of the dmf-t score, compared to the group with a lower education (R^2^ = 0.44 vs. R^2^ = 0.32). This result may be explained by the fact that individuals with a higher education tend to have a more accurate assessment of their own opinions.

Several limitations should be considered when interpreting the findings of this study. First, the study population was recruited from a single region in Lithuania. Although this region is relatively large, comprising about 15% of the country’s population, and does not differ significantly from other regions in socioeconomic terms [58], caution should be exercised when generalizing the results on a national scale. Second, a 71% response rate was achieved in our study, which is generally considered good and adequate to support the strength and validity of the findings. However, a potential bias may still arise if non-respondents differ significantly from respondents in key characteristics. While we were unable to test for such differences, the likelihood is low, as the selected kindergartens were of equal status and their parent communities tend to be highly homogeneous. Third, the study utilized SEM methods to analyze cross-sectional data. As a result, the sequence of events and temporal changes could not be identified. Since the development of caries is chronic and progressive, longitudinal studies are essential for understanding the evolving relationship between parental attitudes and children’s oral health over time, as well as for establishing causality. Additionally, caries is a multifactorial disease [59,60]. While studies analyzing a limited number of factors contribute to the understanding of its etiology, they may not provide a comprehensive picture. In this regard, our study is also limited. However, the factors included in our analysis accounted for a substantial proportion (41%) of the variance in early childhood caries. Finally, our study did not include a section on the children’s diet in the parental questionnaire. Consequently, we were unable to examine the pathway from parental attitudes to childhood dental caries via sugar consumption restrictions. However, based on previous studies, such a pathway is likely relevant, particularly concerning the sugar-snacking component of parental attitudes [10,14].

Despite the limitations mentioned above, the results of this study offer new insights into the relationship between parental attitudes and children’s oral health. These findings may also be valuable in developing intervention strategies for families with preschoolers.

## 5. Conclusions

In children aged 4–7 years, the direct relationship between parental attitudes toward child oral health and dental decay outcomes is very weak. However, this relationship exists indirectly. Its pathway involves regular toothbrushing, parental assistance with toothbrushing, and good oral hygiene, which are critical factors linking parental attitudes to a child’s experience of early caries. Therefore, positive parental attitudes toward oral health can be essential for promoting good oral health skills and the prevention of early dental decay in preschoolers.

## Figures and Tables

**Table 1:** Socio-demographic and clinical characteristics of the sample (N = 302).

Characteristic	Coding	*n*/Mean	%/SD
Sex of children:			
Boys	1	164	54.3
Girls	2	138	45.7
Age of children:			
<5 years	1	151	50.0
≥5 years	2	151	50.0
Mean (SD)		5.18	0.84
Participating parents:			
Fathers	1	40	13.2
Mothers	2	262	86.8
Education level of participating parents:			
Less than college (lower)	1	76	25.2
College or university (higher)	2	226	74.8
Household location:			
Urban area	1	202	66.9
Rural area	2	100	33.1
Family income:			
<2700 EUR/month	1	106	49.3
≥2700 EUR/month	2	109	50.7
Missing		87	
Parental assistance with child’s toothbrushing			
The child brushes their teeth by themself	1	142	47.0
Parents assist with child’s toothbrushing	2	160	53.0
Child’s toothbrushing frequency:			
At least twice a day	1	167	55.3
Once a day	2	119	39.4
Less often than once a day or irregularly	3	16	5.3
Never	4	0	0
Parents’ toothbrushing frequency:			
At least twice a day	1	228	75.5
Once a day	2	64	21.2
Less often than once a day or irregularly	3	10	3.3
Never	4	0	0
Child’s dental visits:			
Regular	1	257	85.1
Irregular	2	45	14.9
Assessment of child’s oral hygiene (OHI):			
Excellent (no plaque)	0	95	33.3
Good	1	87	30.5
Satisfactory	2	68	23.9
Poor (heavy plaque)	3	35	12.3
Mean (SD)		1.14	0.99
Child’s dental caries experience (dmf-t index):			
Minimum (the lowest experience)	0		
Maximum (the highest experience)	20		
Mean (SD)		3.64	3.91

**Table 2:** Descriptive statistics of child’s dental caries experience (dmf-t index), by socio-demographic groups of respondents.

Group of Respondents	N	Mean	Std. Deviation	95% Confidence Interval for Mean		T (300)	*p*-Value
				Lower Bound	Upper Bound		
Sex of children							
Boys	164	3.62	3.99	3.01	4.24	0.067	0.947
Girls	138	3.65	3.82	3.01	4.30		
Age of children							
<5 years	151	3.01	3.98	2.37	3.65	2.801	0.005
≥5 years	151	4.26	3.74	3.66	4.86		
Participating parents							
Fathers	40	3.55	4.46	2.13	4.97	0.149	0.882
Mothers	262	3.65	3.83	3.18	4.11		
Education level of participating parents							
Lower	76	5.24	4.81	4.14	6.34	4.246	<0.001
Higher	226	3.10	3.40	2.65	3.54		
Household location							
Urban area	202	3.26	3.54	2.77	3.75	2.379	0.018
Rural area	100	4.39	4.48	3.50	5.28		
Family income							
<2700 EUR/month	106	4.08	3.89	3.33	4.83	2.074	0.039
≥2700 EUR/month	109	3.00	3.78	2.28	3.72		

**Table 3:** Descriptive statistics of sum score of PACOH scale by socio-demographic groups of respondents.

Group of Respondents	N	Mean	Std. Deviation	95% Confidence Interval for Mean		T (300)	*p*-Value
				Lower Bound	Upper Bound		
Total sample							
	302	122.7	10.0	121.6	123.8		
Sex of children							
Boys	164	123.3	10.1	121.7	124.9	1.166	0.245
Girls	138	122.0	9.8	120.3	123.6		
Age of children							
<5 years	151	123.2	10.5	121.5	124.9	0.877	0.381
≥5 years	151	122.2	9.5	120.7	123.7		
Participating parents							
Fathers	40	123.9	11.0	120.4	127.4	0.840	0.401
Mothers	262	122.5	9.9	121.3	123.7		
Education level of participating parents							
Lower	76	119.3	10.3	117.0	121.7	3.446	0.001
Higher	226	123.8	9.7	122.6	125.1		
Household location							
Urban area	202	124.4	9.1	123.1	125.8	4.286	<0.001
Rural area	100	119.3	10.8	117.1	121.4		
Family income							
<2700 EUR/month	106	120.2	10.0	118.6	122.4	4.241	<0.001
≥2700 EUR/month	109	125.9	8.6	124.3	127.5		

**Table 4:** Spearman’s correlations between study variables.

	Sum Score of PACOH Scale	dmf-t Index	Oral Hygiene Index	Children’s Toothbrushing Frequency	Parents’ Toothbrushing Frequency	Parental Assistance with Toothbrushing
dmf-t index	−0.21 **					
Oral hygiene index	−0.25 **	0.69 **				
Children’s toothbrushing frequency	−0.27 **	0.27 **	0.28 **			
Parents’ toothbrushing frequency	−0.17 **	0.06	0.04	0.37 **		
Parental assistance with toothbrushing	0.25 **	−0.47 **	−0.52 **	−0.20 **	−0.06	
Dental visits	0.05	0.10	0.12 *	0.05	0.02	−0.17 **

Notes: PACOH: Parental Attitudes Towards Child Oral Health; * *p* < 0.05; and ** *p* < 0.01.

**Table 5:** Several estimates of the complete path model: results from AMOS multi-group analyses by socio-demographic variables.

Characteristics	Total Sample (*n* = 302)	Estimates by Socio-Demographic Variables											
		Gender of Children		Age of Children		Parents		Education Level of Parents		Household Location		Family’s Income	
		Boys (*n* = 164)	Girls (*n* = 138)	<5 yrs (*n* = 151)	≥5 yrs (*n* = 151)	Fathers (*n* = 40)	Mothers (*n* = 262)	Lower (*n* = 76)	Higher (*n* = 226)	Urban a. (*n* = 202)	Rural a. (*n* = 100)	Lower (*n* = 106)	Higher (*n* = 109)
**Path**	Standardized measurement weights (β)												
PACOH → Child’s toothbrushing frequency	−0.17 **	−0.15 *	−0.19 *	−0.19 *	−0.15	−0.23	−0.15 ***	−0.05	−0.21 ***	−0.21 ***	−0.09	−0.11	−0.22 *
PACOH → Parents’ toothbrushing frequency	−0.21 ***	−0.22 **	−0.21 *	−0.26 ***	−0.16 *	−0.22	−0.22 ***	−0.30 *	−0.13 *	**−0.08**	−0.33 ***	−0.21 *	−0.13
Parents’ toothbrushing frequency → Child’s toothbrushing frequency	0.33 ***	0.34 ***	0.28 ***	0.32 ***	0.53 ***	0.21	0.35 ***	0.43 ***	0.29 ***	0.30 ***	0.37 ***	0.42 ***	0.25 **
Child’s toothbrushing frequency → Oral hygiene index	0.20 ***	0.18 **	0.22 **	**0.10**	0.30 ***	**−0.04**	0.24 ***	0.20	0.21 ***	0.19 **	0.18 *	0.30 **	0.20 *
Oral hygiene index → dmf-t index	0.52 ***	0.45 ***	0.63 ***	0.48 ***	0.57 ***	0.30 *	0.56 ***	0.37 ***	0.57 ***	0.57 ***	0.44 ***	0.61 ***	0.58 ***
PACOH → Parental assistance	0.24 ***	0.25 ***	0.21 *	0.18 *	0.30 ***	0.19	0.25 ***	0.18	0.21 ***	0.24 ***	0.12	0.17	0.26 **
Parental assistance → Oral hygiene index	−0.47 ***	−0.46 ***	−0.48 ***	−0.49 ***	−0.42 ***	−0.65 ***	−0.44 ***	−0.41 ***	−0.44 ***	−0.40 ***	−0.51 ***	−0.43 ***	−0.41 ***
Parental assistance → Child’s toothbrushing frequency	−0.16 **	−0.13	−0.19 *	−0.13	−0.17 *	−0.14	−0.15 *	−0.14	−0.16 **	−0.11	−0.20 *	−0.20 *	−0.05
Parental assistance → dmf-t index	−0.17 ***	−0.28 ***	**−0.02**	−0.20 *	−0.14 *	−0.46 ***	−0.13 *	−0.22 *	−0.16 **	−0.18 **	−0.20 *	−0.16 *	−0.11
PACOH → dmf-t index	−0.06	−0.12 *	0.04	−0.11	0.01	−0.21 *	−0.03	−0.17	−0.01	−0.02	−0.16 *	−0.02	−0.06
Variable	Squared multiple correlations (R^2^)												
Oral hygiene index	0.30	0.28	0.33	0.27	0.32	0.41	0.30	0.24	0.28	0.22	0.33	0.33	0.23
dmf-t index	0.42	0.46	0.41	0.39	0.43	0.57	0.41	0.32	0.44	0.45	0.58	0.61	0.42

Notes: the significance of the difference from the zero value: * *p* < 0.05, ** *p* < 0.01, and *** *p* < 0.001; underlined and bolded estimates differ significantly (*p* < 0.05) between respondent groups.

## Data Availability

The dataset is available on request from the corresponding author.

## Abbreviations

The following abbreviations are used in this manuscript:

PACOH	Parental Attitudes towards Child Oral Health
OHI	Oral hygiene index
dmf-t	The number of decayed, missing, and filled primary teeth
SEM	Structural Equation Modeling
IFI	Incremental Fit Index
TLI	Turker-Lewis Index
CFI	Comparative Fit Index
RMSEA	Root Mean Square Error of Approximation
